# 3D printing of dual-cell delivery titanium alloy scaffolds for improving osseointegration through enhancing angiogenesis and osteogenesis

**DOI:** 10.1186/s12891-021-04617-7

**Published:** 2021-08-27

**Authors:** Heng Zhao, Shi Shen, Lu Zhao, Yulin Xu, Yang Li, Naiqiang Zhuo

**Affiliations:** Department of Department of Bone and Joint, Affiliated Hospital of Southwest Medical University, 646000 Luzhou, People’s Republic of China

**Keywords:** 3D printed, Titanium alloy implant, Angiogenesis, Osteogenesis, Osseointegration

## Abstract

**Background:**

The repair of large bone defects is a great challenge for orthopedics. Although the development of three-dimensional (3D) printed titanium alloy (Ti6Al4V) implants with optimized the pore structure have effectively promoted the osseointegration. However, due to the biological inertia of Ti6Al4Vsurface and the neglect of angiogenesis, some patients still suffer from postoperative complications such as dislocation or loosening of the prosthesis.

**Methods:**

The purpose of this study was to construct 3D printed porous Ti6Al4V scaffolds filled with bone marrow mesenchymal stem cells (BMSC) and endothelial progenitor cells (EPC) loaded hydrogel and evaluate the efficacy of this composite implants on osteogenesis and angiogenesis, thus promoting osseointegration.

**Results:**

The porosity and pore size of prepared 3D printed porous Ti6Al4V scaffolds were 69.2 ± 0.9 % and 593.4 ± 16.9 μm, respectively, which parameters were beneficial to bone ingrowth and blood vessel formation. The BMSC and EPC filled into the pores of the scaffolds after being encapsulated by hydrogels can maintain high viability. As a cell containing composite implant, BMSC and EPC loaded hydrogel incorporated into 3D printed porous Ti6Al4V scaffolds enhancing osteogenesis and angiogenesis to repair bone defects efficiently. At the transcriptional level, the composite implant up-regulated the expression levels of the osteogenesis-related genes alkaline phosphatase (*ALP*) and osteocalcin (*OCN*), and angiogenesis-related genes hypoxia-inducible factor 1 alpha (*HIF-1α*), and vascular endothelial growth factor (*VEGF*).

**Conclusions:**

Overall, the strategy of loading porous Ti6Al4V scaffolds to incorporate cells is a promising treatment for improving osseointegration.

## Introduction

Large bone defects caused by trauma, joint revision, reconstruction after bone tumor resection, and congenital deformities are very common in orthopedics and can easily lead to serious outcomes. At present, the clinical treatment of bone defects in orthopedics is mainly used autologous bone grafts, allogeneic bone grafts, as well as artificial bone substitutes. These strategies fill the defects and create bridges for mechanical support and bone regeneration [1]. However, the disadvantages of autologous bone graft (e.g., surgical pain, lack of donors, donor-site complications) and allogeneic bone graft (e.g., immune rejection and potential disease transmission) have boosted the exploration of novel bone substitutes [2].

Titanium alloy (Ti6Al4V) is one the most widely used artificial bone substitutes, because of its biocompatible and suitable mechanical properties. However, due to its biological inertness, leading these “inanimate” materials can only be used as fixation materials support the bone defects, and cannot form good osseointegration between the prosthesis and host bone, consequently [3]. Previous researches have demonstrated that controllable interconnected porous structures are superior to provide stability at the early stage, induce bone regeneration, and promote osseointegration [4, 5]. The emergence of three-dimensional (3D) printing technology enables surgeon to fabricate Ti6Al4V prostheses with arbitrary shape matching the bone defects, controllable pore shapes and porosity, good pore connectivity, as well as the clinical applicability [6]. 3D printed metal implants have already been used in clinical practice and achieve good prognosis [7–9]. However, some patients with limited osteogenesis, such as osteoporosis, suffer serious complications, e.g., displacement or loosening of the implants, after prosthesis implantation due to lack of osseointegration. Therefore, finding a strategy that can effectively promote bone formation, accelerate the osseointegration, and avoid the occurrence of the above postoperative failure is a goal worth exploring.

Bone marrow mesenchymal stem cells (BMSC) is widely used as seed cells in tissue engineering and regenerative medicine [10]. Various researches have indicated that BMSC have great potential for enhancing bone regeneration, owing to its high capacity for self‑renewal and multi differentiation potential. Consequently, BMSC are regarded as an ideal seed cell for promoting osteogenesis in bone tissue engineering [11]. In addition, a series of surface modification strategies have also been used to induce osteogenesis and osseointegration of the porous titanium scaffolds [12, 13]. Although it is recognized that the use of additional BMSC incorporation or prosthetic surface modification to treat bone defects can effectively accelerate bone formation, the size of the regenerated bone has always been a restriction for sufficient bone repair, mainly because of the absence of blood vessels in the grafts [14, 15]. Since abundant angiogenesis is critical to provide sufficient nutritional support, active osteoblast function, and accelerate new bone formation. Therefore, the lack of attention to angiogenesis after the implantation of porous titanium implants is a critical factor leading to the failure of long-term fixation [16]. Osteogenic cells migration and growth into the porous implants need affluent blood and oxygen support for direct osteogenesis [17]. The early formation of neovascularization in the bone tissue around the implant is essential for the rapid induction of osteogenesis and osseointegration, which can effectively improve operative success rate after implantation [18]. Therefore, a dual-functional composite porous implant with both osteogenesis and angiogenesis characteristics is needed for fixation and long-term survival of prosthesis. Endothelial progenitor cells (EPC) are a subgroup of pluripotent hematopoietic stem cells that can proliferate and migrate to the site of endothelial injury and differentiate into vascular endothelial cells to facilitate neovascularization [19]. Additionally, previous studies have shown that EPC may in favor of bone regeneration in fracture healing [20, 21]. Therefore, EPC is considered to be a crucial factor in promoting angiogenesis and accelerating bone formation after bone injury.

With regard to the respective characteristics of BMSC and EPC, Seebach et al. have attempted to seed BMSC and EPC onto beta-tricalcium phosphate granules to promote early angiopoiesis and bone formation in critical-sized bone defects [22]. However, it is difficult for 3D printed porous Ti6Al4V scaffolds which can bear loading and induce bone ingrowth to adhere enough cells to the surface for subsequent transplantation. Therefore, the introduction of a cellular carrier to distribute the active cells evenly and stably in the pores of the scaffold is urgently needed. Hydrogels are consisted by 3D network of hydrophilic polymers. Nowadays, hydrogels are not only regarded as drug delivery systems, but also widely used in 3D cell culture system because of its similar properties with natural extracellular matrix [23–25].

In the present study, poloxamer 407, a thermosensitive hydrogel, was used as a cell carrier to incorporate BMSC and EPC into the pores of the 3D printed Ti6Al4V porous scaffolds for the study of osseointegration. Poloxamer 407, is a kind of polymer which is sensitive to temperature change. That is, when the ambient temperature is lower than the critical transition temperature, the polymer exhibits solution state, and the semisolid gel state will appear when the temperature is higher than the critical transition temperature. The formation mechanism of polymer is mainly the reversible change of polymer dispersion or conformation under the action of external temperature, which leads to the change of physical state of polymer, that is, the transformation from solution state to semisolid gel state [26]. The most attractive feature of poloxamer 407 is its reversible thermo-responsive property, allowing it to undergo gelation near body temperature (∼37 °C) and remain at the site of implantation as a continuous drug delivery device. In low temperature conditions (< 15–25 °C depending on the polymer weight), poloxamer 407 exists in the form of solution, during which it can be loaded with therapeutics for later release from its gel state [26]. Thanks to its reversible thermo-responsive nature, biocompatibility, high bioavailability, poloxamer 407 hydrogel has been used widely in the drug delivery for sustained release and 3D cell culture system [27, 28]. In this study, due to the addition of cells incorporation by poloxamer 407 hydrogel, we speculate the porous scaffolds could have a superior performance to promote angiogenesis and osteogenesis, thus enhancing osseointegration.

## Materials and methods

### Materials

Ti6Al4V powder was obtained from AK Medical (Beijing, China). Poloxamer 407 was supplied by Bayee Chemical Co., Ltd. (Hangzhou, China). Rabbit endothelial progenitor cells (BFN60810500) was purchased from ATCC (Manassas, VA, USA) and Rabbit BMSCs (RBXMX-01001) was supplied by Cyagen Biosciences (Guangzhou, China). Low Glucose Dulbecco’s Modified Eagle’s Medium (LG-DMEM), fetal bovine serum (FBS), and streptomycin double antibody were supplied by Gibco (Grand Island, NY, USA). Cell Counting Kit‑8 (CCK‑8) assay was supplied by (Dojindo, Shanghai, China) and Calcein-AM/PI Kits was purchased from Beyotime Co., Ltd. (Shanghai, China). Microfil (MV122, Flow Tech, Carver, MA, USA) was used for microangiography. RNA and the Revert Aid First Strand cDNA Synthesis Kit, and SYBR Premix Ex TaqTM kit were supplied by TaKaRa (Dalian, China). Trizol Reagent was obtained from Invitrogen (CA, USA).

### Preparation and characterization of 3D printed scaffolds

Cylindrical 3D printed Ti6Al4V porous scaffolds, which was 6 mm diameter and 8 mm height, with pre-designed pore sizes about 600 μm and porosity about 70 % were manufactured layer-by-layer by an EBM system (Arcam Q10, Sweden). Briefly, the 3D model date was designed and imported into the UG NX6.0 system (Unigraphics Solutions, US). Medical-graded Ti6Al4V powder with an average diameter of 45–105 μm were used as basic material. The maximum scanning speed of electron beam was 8000 m s^− 1^ and the accuracy of printing is ± 0.4 mm. Finally, all samples were cleaned in Powder Recovery System and washed in acetone, ethanol, and distilled water with ultrasonic machine in turn for 30 min, respectively, before cell experiments and animal procedures [29].

To demonstrate whether the parameters of the prepared scaffolds, such as pore size and porosity, are the same as those of the pre-designed model, we performed characterization tests on the scaffolds. The porosity of prepared porous scaffolds was measured by a SkyScan 1076 scanner Microcomputed Tomography (Micro-CT, Bruker, Kontich, Belgium). In addition, in order to detect the average pore diameter of these Ti6Al4V scaffolds, the microstructure of samples was photographed by a SIGMA500 scanning electron microscope (SEM, ZEISS, Oberkochen, Germany), and pictures were quantitative analysis by Image J software (NIH, Bethesda, MD, USA).

### Preparation of cells-loaded hydrogel and incorporation into porous scaffolds

Poloxamer 407 hydrogel was prepared *via* one pot mix the Poloxamer powder with 0.01 M phosphate buffer saline (PBS, pH = 7.4) at 25 % w/w. Poloxamer 407 powder was completely dissolved at 4 °C in to obtain a transparent and uniform solution.

Then, a certain amount of BMSC and/or EPC were incorporated into the hydrogel solution to prepare a cell-loaded hydrogel. The porous scaffolds were inserted in a chamber of the 96-well culture plate, and then corresponding volume of hydrogel solution or cell-loaded hydrogel solution was injected into the chamber to fill the porous scaffolds at 15 °C. The hybrid scaffolds were promptly warmed to 37 °C to transfer the hydrogel from solution into gel. Finally, take out the composite scaffold carefully from the chamber by an eye tweezer for subsequent cellular and animal experiments.

### Biocompatibility and cell viability

In order to study the biocompatibility of the 3D printed porous Ti6Al4V scaffolds (namely empty scaffolds, abbreviated as eTi) and poloxamer 407 hydrogel incorporated porous Ti6Al4V scaffolds system (abbreviated as hTi), CCK-8 assay was conducted. In brief, BMSC and EPC were seeded in 48 well plates at a density of 5 × 10^4^ cells per well. After the cells were adhered, eTi and hTi were put into the plates. In parallel with the porous scaffolds assessment, control groups (abbreviated as Con) were cells cultured without the scaffolds. Cells were incubated in a humidified incubator at the atmosphere of 37 °C and 5 % CO_2_, and the LG-DMEM were replaced every 3 days. The cell proliferation in each well was detected at 1, 3, and 5 days of culture. That is to say, at every time interval, the LG-DMEM in each well was replenished with fresh medium containing 10 % CCK-8 solution. After incubation for 2 h in the incubator, 100 µl solution of each sample was transferred to the 96-well plate for absorbance detection by a Microplate Reader (Multiskan EX, Thermo Fisher Scientific, MA, USA) at 450 nm.

In addition, in order to study the proliferation and cell viability of BMSC and EPC within the hTi, cells were resuspended in poloxamer 407 solution and compounded with the Ti6Al4V scaffolds embedded in a 96 well plate, then the composite system was formed at 37 ℃. BMSC/hTi represents the scaffolds filled with 5 × 10^5^ BMSC-loaded hydrogel, EPC/hTi means the scaffolds filled with 5 × 10^5^ EPC-loaded hydrogel, and Dual/hTi indicates the scaffolds filled with 5 × 10^5^ BMSC and EPC-loaded hydrogel. The cell proliferation of each sample was detected after 1, 3, and 5 days of culture. Furthermore, to evaluate the cell viability of BMSC and EPC within the hTi, Live/Dead cell staining was conducted through the Calcein-AM/PI Kit on the basis of the manufacturer’s procedure after 3 days of culture. The fluorescent images were photographed by confocal laser scanning microscope (CLSM) (FV1000, Olympus, Japan). And then, the percentage of live cell, namely, cell survival rate, was calculated according to the proportion of live cells and total cells by Image J software.

### Animal surgical procedures

The *in vivo* animal study was approved by the Animal Care and Use Ethics Committee of Affiliated Hospital of Southwest Medical University. New Zealand white rabbits (*n* = 30, 5-month-old, female) were used to prepare lateral femoral condyle defect models to detect the therapeutic effects of EPC and BMSC-loaded 3D printed porous Ti6Al4V scaffolds in inducing angiogenesis and osteogenesis. Briefly, implantation operation was conducted under general anesthesia with 3 % (w/v) pentobarbital at a dosage of 50 mg/kg. A 1.5 cm longitudinal incision was made on the lateral condyle of the distal femur after skin preparation and local disinfection. After separating the tissue layer by layer and exposing the bony surface, cylindrical bone defects (6 mm diameter, 8 mm depth) were produced by an aseptic drill. And then, different composite scaffolds were implanted into the defects, and the incisions were then sutured layer by layer with absorbable sutures. Penicillin at a dosage of 1.5 mg/kg was administrated by intramuscular injection for 3 consecutive days for prevention of postoperative infection. *In vivo* experiments were divided into five groups (*n* = 6), namely, empty Ti6Al4V scaffolds (abbreviated as eTi Group), composite Ti6Al4V scaffolds containing unloaded poloxamer 407 hydrogel (abbreviated as hTi Group), composite Ti6Al4V scaffolds containing EPC-loaded poloxamer 407 hydrogel (abbreviated as EPC/hTi Group), composite Ti6Al4V scaffolds containing BMSC-loaded poloxamer 407 hydrogel (abbreviated as BMSC/hTi Group), and composite Ti6Al4V scaffolds containing EPC and BMSC-loaded poloxamer 407 hydrogel (abbreviated as Dual/hTi Group).

Twelfth weeks after scaffolds implantation, rabbits were euthanized by excessive dose of 3 % (w/v) pentobarbital (120 mg/kg). The femur samples were collected after removing the surrounding soft tissue, and then preserved in 4 % polyformaldehyde solution or -80 ℃ for subsequently osteogenesis and angiogenesis evaluation.

### Microangiography

At 12 weeks after implantation surgery, the rabbits used for microangiography under anesthesia according to previous study [30]. In brief, a midline abdominal incision is performed to expose the abdominal aorta and postcava. Both postcava and abdominal aorta were cut and ligated at the proximal end. Subsequently, an infusion tube was inserted into the abdominal aorta, and heparin sodium (50 IU/mL) contained normal saline (about 1 L) was applied to flush the blood vessels of the lower limbs through an electric syringe. And then, about 0.5 L 10 % formaldehyde was used to fix the lower limb vessels through the abdominal aorta. Finally, 50 mL of Microfil® silicone rubber injection compound was injected by the abdominal aorta to perfuse lower limb vessels (2 mL/min).

### Micro-CT evaluation

To detect the therapeutic effects of bone ingrowth and vascularization, the femur samples were scanned by Micro-CT. According to the original imaging pictures, 3D reconstruction was conducted to investigate the bone formation around the scaffolds. Subsequently, quantitative morphometric analysis of the region of interest (ROI) was performed to evaluate the quality of bone ingrowth. In addition, in order to demonstrate the angiogenesis in different samples, the area of 2 mm around the microangiography specimen was used as the ROI to evaluate the vascular regeneration.

The assessment of newly formed bone quality and blood vessel volume were represented by bone volume/total volume (BV/TV, %), trabecular thickness (Tb.Th, mm), trabecular number (Tb.N, 1/mm), trabecular separation (Tb.Sp, mm), as well as blood vessel volume/total volume (BVV/TV, %), respectively.

### Histological evaluation

The samples of distal femur with scaffolds were dehydrated and then embedded in methyl methacrylate. Subsequently, the samples were prepared to be 150–300 μm thick sections through a transverse saw cuts and a polishing machine (EXAKT Apparatebau, Norderstedt, Germany). And then, the thick sections were grinded and polished to about 50 μm, and stained by Masson’s Trichrome on the basis of the manufacturer’s procedure to observe the bone ingrowth into the porous scaffolds.

### Quantitative real-time PCR

After the rabbits were euthanized at 12 weeks after implantation, bone tissue samples were collected from the surface and the pores of the scaffolds. In a liquid nitrogen contained mortar, the bone tissue was ground into powder. Total cellular RNA was extracted from the tissues, and cDNA was synthesized. The transcription levels of alkaline phosphatase (*ALP*), osteocalcin (*OCN*), hypoxia-inducible factor 1 alpha (*HIF-1α*), and vascular endothelial growth factor (*VEGF*) were measured. GAPDH was used to normalize the transcriptional level. The primer sequences were displayed in Table 1.

**Table 1:** Primer sequences of genes

Gene subtype	Oligonucleotide Primers (5’-3’)
ALP	F: ATC GGA CCC TGC CTT ACC R: CTC TTG GGC TTG CTG TCG
OCN	F: AGCCACCGAGACACCATGAGA R: AGCCACCGAGACACCATGAGA
HIF-1α	F: GAACGTCGAAAAGAAAAGTCTCG R: CCTTATCAAGATGCGAACTCACA
VEGF	F: GAGGAGCAGTTACGGTCTGTG R: TCCTTTCCTTAGCTGACACTTGT
GAPDH	F: CAATGACCCCTTCATTGACC R: TGGACTCCACGACGTACTCA

### Statistical analysis

All results were represented by mean ± standard deviation (SD). Comparisons among groups were analyzed with one‑way ANOVA followed by Tukey’s post hoc test by SPSS 19.0 (SPSS Inc., Chicago, USA). A factor of *p < 0.05* was regarded as statistically significant. All experiments were repeated independently at least 3 times.

## Results and discussion

### Characterization of scaffolds

**Fig. 1:**
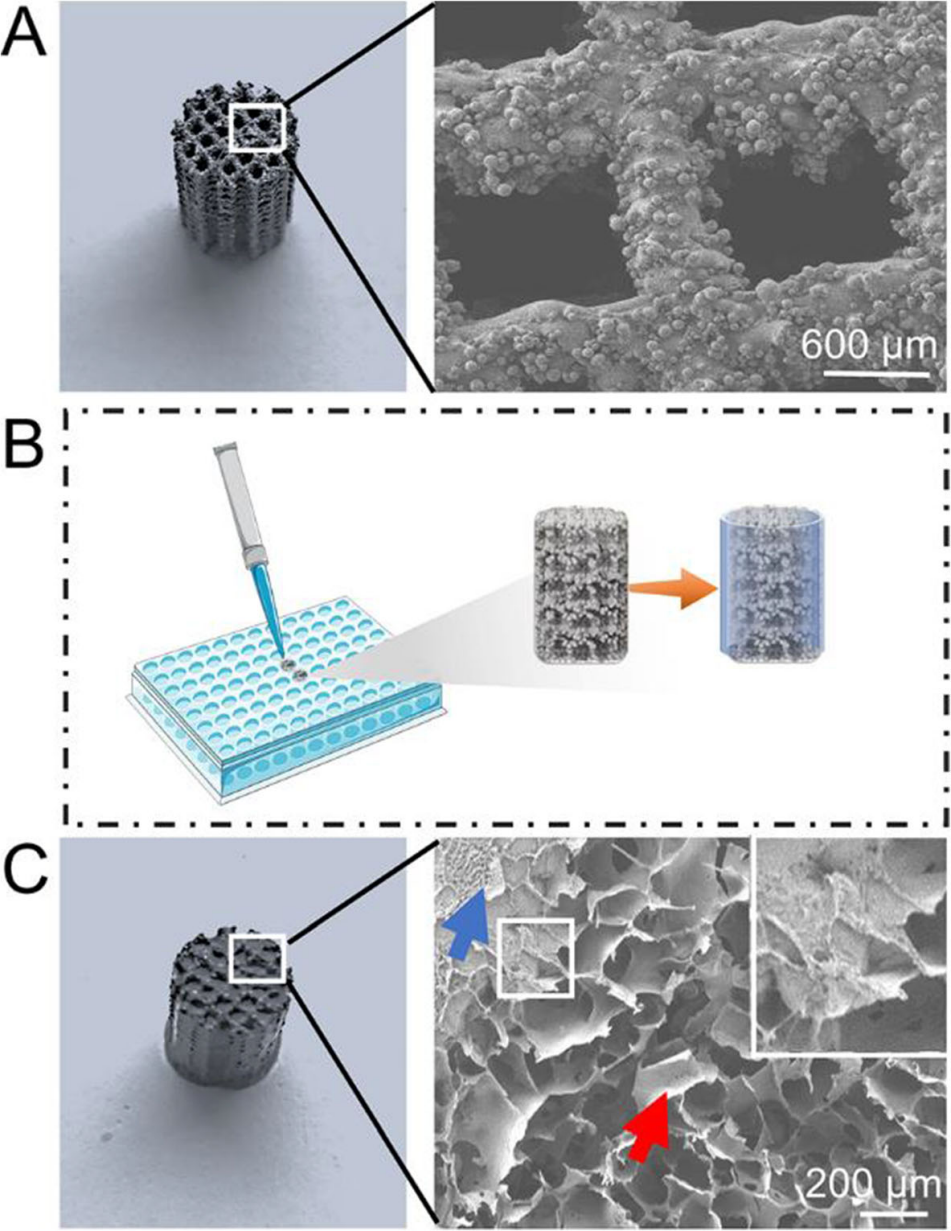
**A** Gross view and SEM image of empty porous scaffold. **B** Schematic diagram of poloxamer 407 hydrogel filled scaffolds. **C** Gross view and SEM image of hydrogel incorporated scaffold. After hydrogel incorporation, the scaffolds (blue arrow) were completely filled with interconnected hydrogel networks (red arrow)

According to the protocols, the Ti6Al4V porous scaffolds were prepared by 3D printing technology successfully. In order to detect that whether the parameters of the scaffolds were in line with the pre-design, Micro-CT was used to measure the porosity and SEM was also carried out to observe the pore size distribution. The results demonstrated that the porosity of the porous scaffolds was 69.2 ± 0.9 %. SEM image of the scaffold was displayed in Fig. 1A, and then quantitative analysis by Image J software indicated the average pore size of the scaffolds was 593.4 ± 16.9 μm. In general, the parameters (porosity and pore size) of the printed scaffolds are consistent with the pre-design model (70 % and 600 μm, respectively).

The osseointegration ability of the bone tissue engineering scaffolds not only depends on the bone conductivity of the material itself, but also is associated with the pore distribution, porosity and pore size of the implants. Increasing the porosity and surface area of the implants to a certain extent can increase the initial stability and coefficient of friction between the bone and scaffolds, thereby reducing micro-motion, inducing bone ingrowth, and accelerating osseointegration after implantation [31]. For an ideal orthopedic porous implant, the porosity should be higher than 50 %, especially in the range of 65–75 %, and its structure and machinery should be similar to human trabecular bone. As for pore size, it should be between 300-700-µm, this range is conducive to the adhesion, proliferation and differentiation of osteoblasts [32, 33]. Therefore, as a bone tissue engineering implant, the porosity and pore size of the porous Ti6Al4V scaffolds in this study were considered to have ideal osteoinductive parameters.

In addition to ideal porosity and pore size, the structure of the scaffold’s pores also has an important impact on osteogenesis and vascularization after implantation. The structure of internal interconnection of the porous implants were also in favor of oxygen and nutrient exchange [34]. Moreover, the interconnected porous structure with internal perforations facilitates mesenchymal stem cells adhesion, proliferation, and differentiation, and provides a rich interface bonding area for blood vessel formation, bone ingrowth and osseointegration. Finally, a long-term stable outcome is achieved, with the implant and bone forming a biologically fixation [35, 36]. Regarding the range of porosity that is conducive to blood vessel formation, previous studies believed that the porosity of 60-70 % is ideal, which can promote the exchange of oxygen, nutrients and metabolite exchange, and benefit cell penetration, and can better induce vascularization [37, 38]. Previous researches have demonstrated that porous scaffolds with a pore size > 300 μm could benefit oxygen and nutrients penetrate into the internal pores of the scaffolds and induce angiogenesis [37, 39]. Herein, the interconnected 3D printed porous Ti6Al4V implants with the average porosity and pore size were 69.2 ± 0.9 % and 593.4 ± 16.9 μm, respectively, was sufficient to induce bone ingrowth and promote vascularization. Generally, 3D printing technology offers a meaningful approach for manufacturing high-precision customized implants with pre-determined parameters of porosity and pore size.

Schematic diagram of poloxamer 407 hydrogel incorporated scaffolds was displayed in Fig. 1B. After permeation with the hydrogel, the original pores of the Ti6Al4V scaffolds were filled with interconnected hydrogel networks ranging from 100 to 300 μm (Fig. 1C), which showed that the composite implant system was constructed successfully.

### Biocompatibility and cell viability

**Fig. 2:**
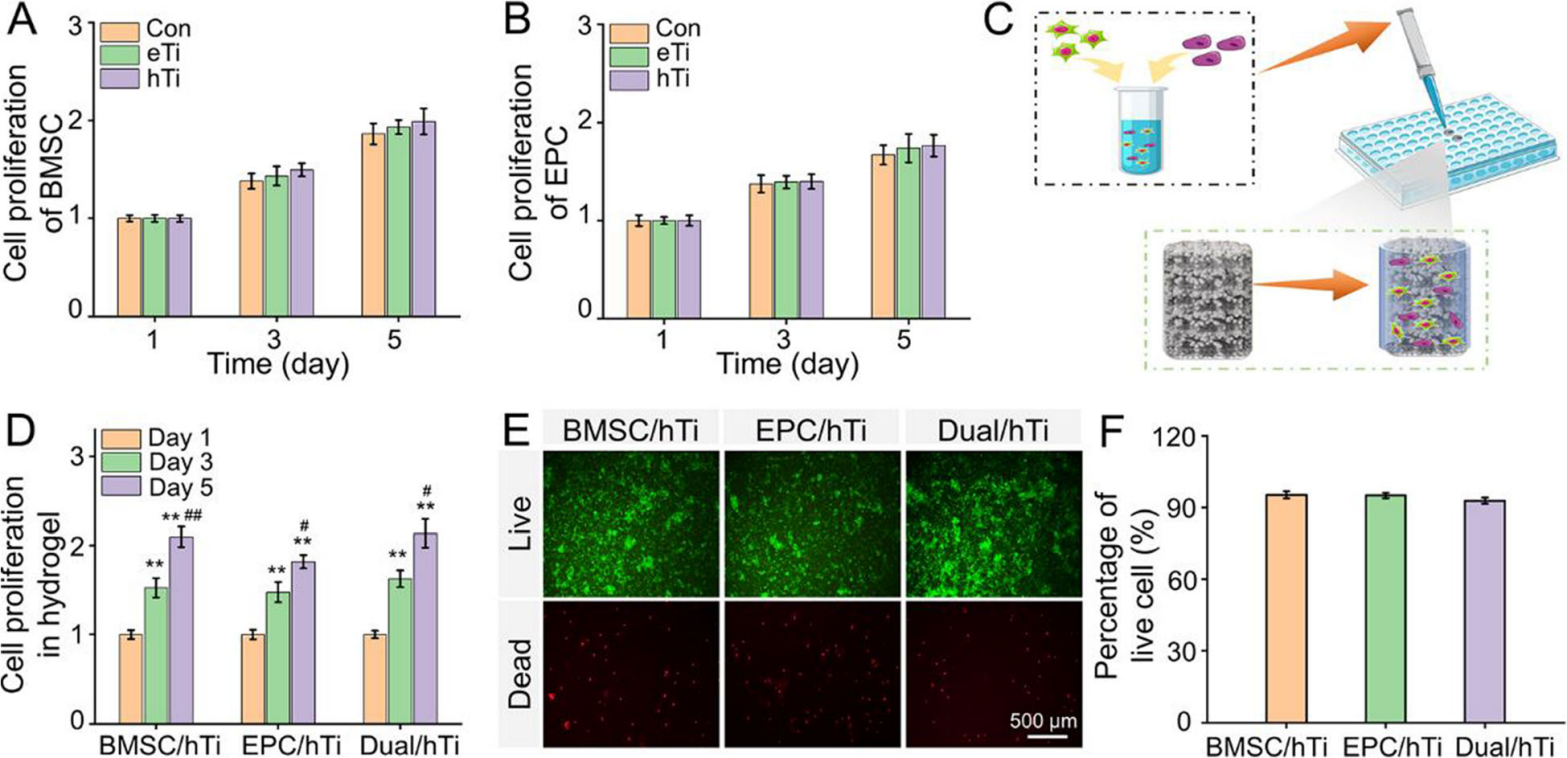
**A** BMSC proliferation and **B** EPC proliferation cultured with Con, eTi, and hTi scaffolds at 1, 3, and 5 days. **C** The schematic diagram of the cell-loaded hydrogel incorporated scaffolds. **D** BMSC and/or EPC proliferation cultured within hTi scaffolds at 1, 3, and 5 days. **E** Calcein AM/PI staining of live cells (green) and dead cells (red) of BMSC/hTi, EPC/hTi, and Dual/hTi groups. **F** Quantitative analysis of cell survival rate (**p < 0.01, compared with the Day 1; ^#^*p* < 0.05 and ^##^*p* < 0.01 compared with the Day 3)

The biocompatibility and of the BMSC and EPC co-cultured with eTi scaffolds and hTi scaffolds were detected by CCK-8 (Fig. 2A and B). BMSC and EPC showed a good proliferation trend when co-cultured in different scaffold samples at all time points. The cell proliferation rates of the BMSC and EPC cultured with eTi and hTi scaffolds were nearly indistinguishable from those of the Con group on the 3rd and 5th day after culture. These results indicated that both porous scaffolds and hydrogel incorporated porous scaffolds have good biocompatibility to cells.

Furthermore, BMSC and/or EPC loaded hydrogel was filled in the porous scaffolds to evaluate the proliferation of these cells. The schematic diagram of the cell-loaded hydrogel incorporated scaffolds is shown in Fig. 2C. As shown in Fig. 2D, with the extension of culture time, the cells increased significantly from day 1 to day 5 within hTi scaffolds. They were displayed simultaneously in BMSC/hTi, EPC/hTi, and Dual/hTi groups, that the number of cells on the 3rd and 5th day was significantly higher than that on the 1st day (*p* < 0.01), and the cell proliferation on the 5th day was also significantly increased than that on the 3rd day (*p* < 0.05). Different cells seeded within hTi scaffolds were stained by Calcein AM/PI to study the cell survival. As displayed in Fig. 2E, the fluorescence images clearly demonstrated that the cells in each group maintained an excellent viability. After 3 days, the viability of BMSC/hTi, EPC/hTi, and Dual/hTi groups were 95.24 ± 1.48 %, 94.99 ± 1.156 %, and 92.79 ± 1.27 %, respectively (Fig. 2F).

The proliferation ability and viability of bone repair related cells in specific microenvironments are crucial for bone generation. Especially for orthopedic implants incorporating with exogenous cells, the proliferation and survival of loaded-cells greatly determines the therapeutic efficacy after implantation [40, 41]. Our results indicated that the poloxamer 407 hydrogel incorporated Ti6Al4V scaffolds were not toxic to the cells, and maintained BMSC and EPC proliferation with good cell viability. BMSC and EPC have the ability to multiply proliferation in this scaffold/hydrogel complex, which greatly expands the number of cells, and provides sufficient primitive cells for subsequent osteogenic differentiation and angiogenic differentiation. Due to lack of sufficient interface biological activity, it is difficult for 3D printed Ti6Al4V scaffolds to adhere enough cells for *in vivo* transplantation. However, this strategy of encapsulating cells in hydrogel, a 3D cell culture system, and then filling into the pores of porous scaffolds provides a potential solution for 3D printing technology combined with cell therapy, and is expected to endow biologically inert titanium alloys with good angiogenesis and osteogenesis properties.

### Evaluation of new bone formation and ingrowth

**Fig. 3:**
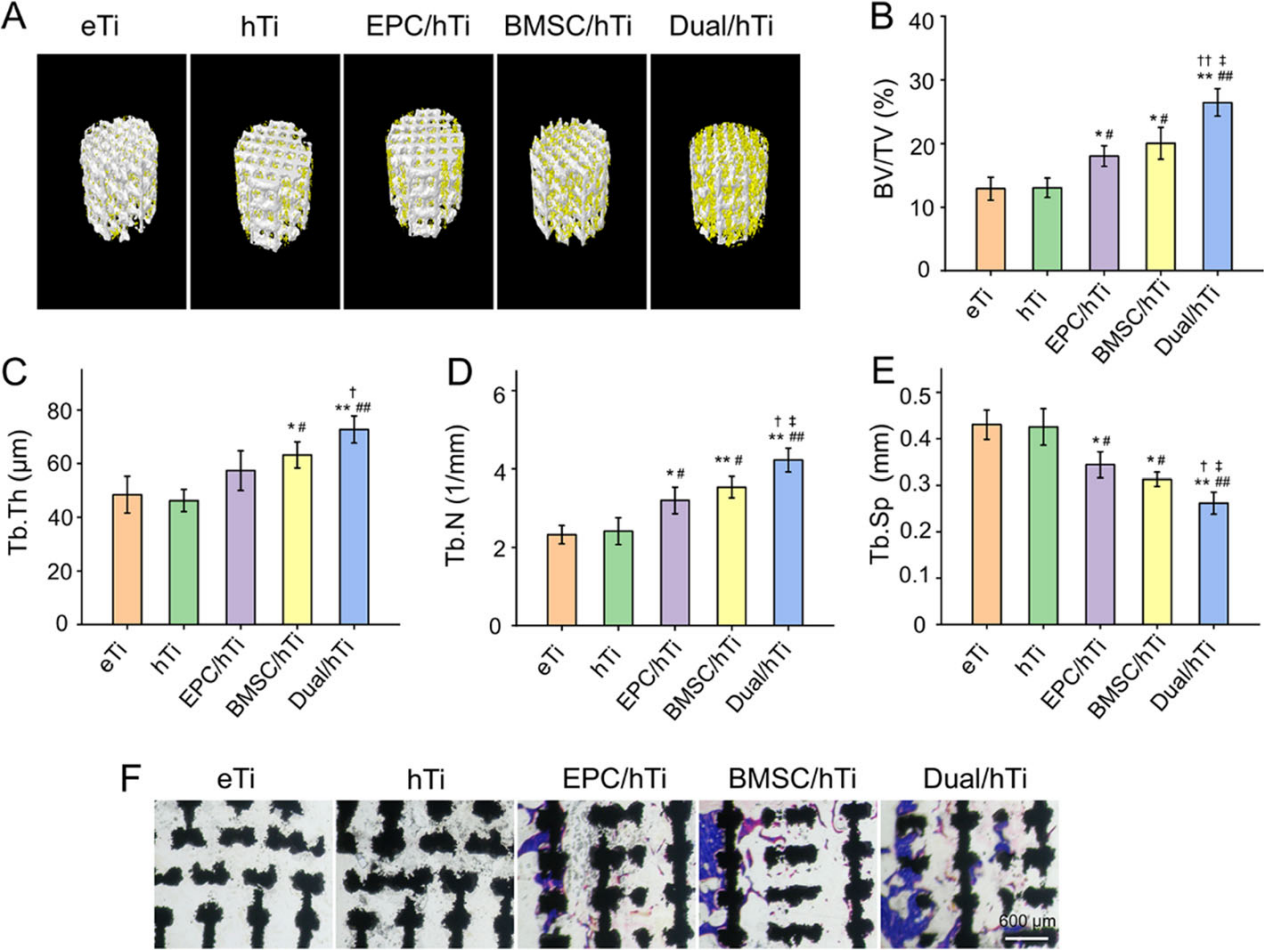
**A** 3D reconstruction images of porous scaffolds after 12 weeks of implantation. Newly formed bone is indicated in yellow, and the scaffolds are seen in white. **B** Bone volume/total volume (BV/TV, %). **C** Trabecular thickness (Tb.Th, mm). **D** Trabecular number (Tb.N, 1/mm). **E** Trabecular separation (Tb.Sp, mm) parameters analysis according to Micro-CT after 12 weeks of implantation. **F** Representative Masson staining images of new bone in and around the porous scaffolds (*p < 0.05, **p < 0.01 compared with the eTi Group; ^#^*p* < 0.05, ^##^*p* < 0.01 compared with the hTi Group; ^†^*p* < 0.05, ^††^*p* < 0.01 compared with the EPC/hTi Group; ^‡^*p* < 0.05 compared with the BMSC/hTi Group)

Bone regeneration and ingrowth into the porous implants was evaluated by Micro-CT. Figure 3A displayed the 3D reconstructed pictures of the scaffolds and surrounding bone tissues. The spatial distribution of the regenerated bone indicated that bone regeneration of the eTi Group and hTi Group were limited, EPC/hTi Group and BMSC/hTi Group were medium, and Dual/hTi Group was abundant. Subsequently, parameters of BV/TV, Tb.Th, Tb.N, and Tb.Sp were used to quantitatively analyze the quality of regenerated bone, according to the Micro-CT results. BV/TV values have been widely applied to quantitatively evaluate the regenerated bone mass [42]. In this study, the BV/TV values of eTi Group, hTi Group, EPC/hTi Group, BMSC/hTi Group, and Dual/hTi Group were 12.92 ± 1.81 %, 13.0 ± 1.52 %, 18.05 ± 1.62 %, 20.07 ± 2.51 %, and 26.46 ± 2.14 %, respectively (Fig. 3B). The BV/TV results were consistent with 3D reconstruction, namely, EPC/hTi Group and BMSC/hTi Group showed more bone formation than eTi Group and hTi Group (*p* < 0.05), and Dual/hTi Group achieved the most superior bone regeneration outcome compared to the other four groups (*p* < 0.05 or *p* < 0.01). Similarly, Dual/hTi Group was provided with the highest Tb.Th and Tb.N values (Fig. 3C and D) and the lowest Tb.Sp among these 5 experimental groups (Fig. 3E). In general, according to the Micro-CT results, EPC-loaded or BMSC-loaded scaffolds significantly enhanced bone regeneration compared with the cell free scaffolds, especially for scaffolds loaded with EPC and BMSC, simultaneously.

Osteointegration between implants and surrounding host bone is critical for bone repair, which including a series of complex biological processes. A functional prosthesis should firmly integrate and combine with the surrounding host bone tissue, thereby reducing the risk of prosthesis displacement and loosening after surgery, and reconstructing the function of bone and limb [33, 43]. In order to observe the osteointegration effect, the undecalcified bone sections were prepared and stained. The results of histological examination were consistent with that of Micro-CT scanning. Representative images were exhibited in Fig. 3F. There was just rare bone regeneration around the scaffolds in eTi Group, hTi Group. However, in EPC/hTi Group and BMSC/hTi Group, more bone tissue was formed and combined with the micropores on the surface of the scaffolds. This phenomenon of bone ingrowth was more obvious in the Dual/hTi Group, regenerated bone tissue not only almost completely integrated with the surface pores of the scaffolds, but also grew into the internal pores.

Sufficient bone formation and ideal osseointegration between implants and surrounding bone is crucial for stability during bone remodeling, which is closely associated with the differentiation, maturation, and mineralization of BMSC [2, 44]. During the healing process, local transplantation BMSC could improve the osteogenic microenvironment in the defects, thus, to promote the progression of bone formation [45]. Therefore, BMSC loaded scaffolds are regarded as a promising tissue engineering strategy to promote repair of bone defects [13].

### Evaluation of neovascularization

When cells were loaded into the hydrogels, the nanochannels in the hydrogels have a positive effect on the exchange of oxygen and nutrients, thereupon then promoting cell growth and communication, and further benefiting bone repair [34, 46]. However, there are limited research has focused on hydrogels as cell carriers or drug delivery systems to incorporate into 3D printed porous titanium implants to form a composite implant. In spite of BMSCs, exogenous BMP-2, as well as VEGF were also incorporated into porous implants to achieve a long-term survival of prosthesis [47, 48]. However, these studies unilaterally promoted bone regeneration but limited to the comprehensive consideration of the synergistic role of BMSC and EPC in osteogenesis and vascularization, therefore they could not obtain the optimal effect of osseointegration.

**Fig. 4:**
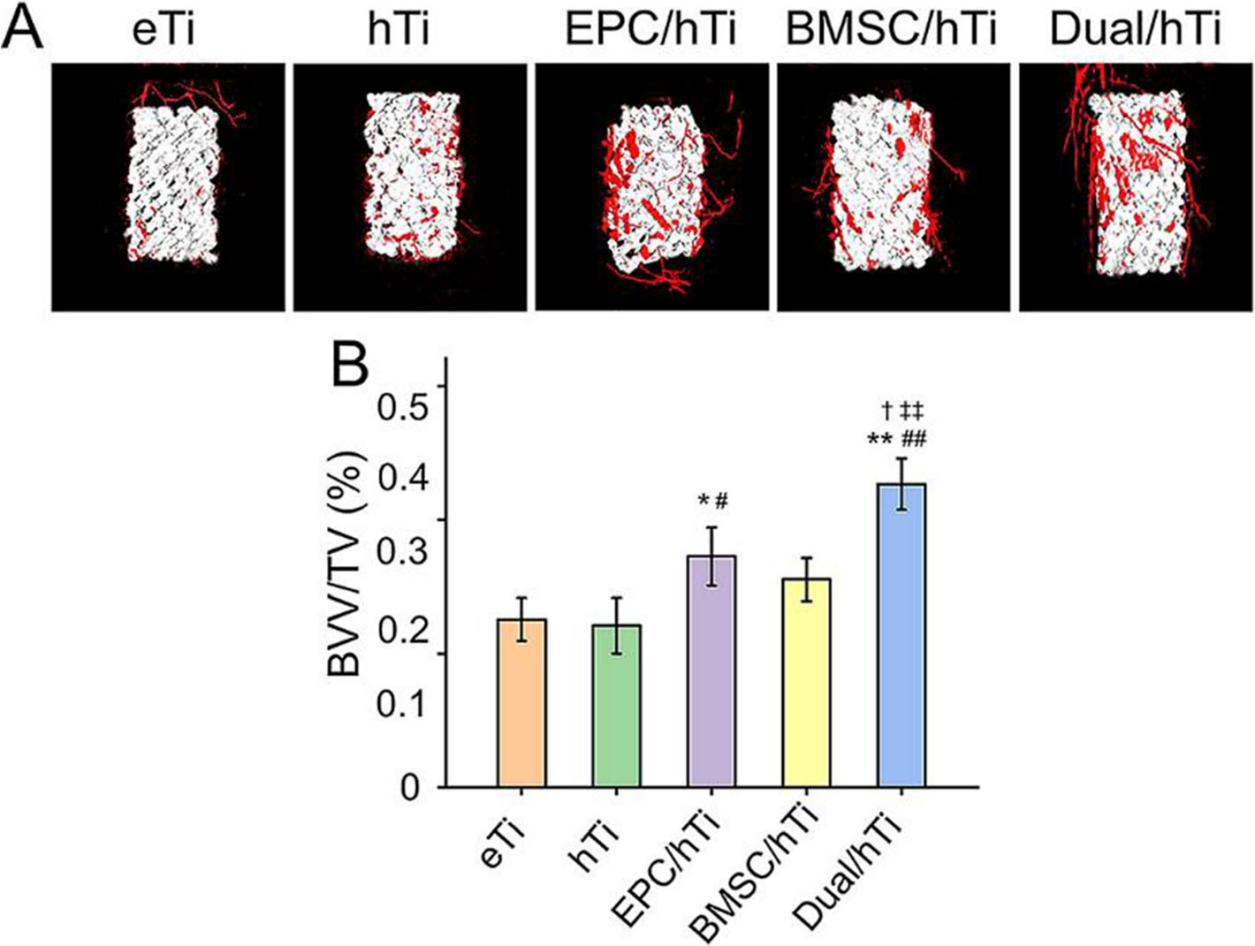
**A** Microangiography images of newly formed blood vessel around the scaffolds. **B** Percentages of BVV/TV in these scaffolds was calculated (*p < 0.05, **p < 0.01 compared with the eTi Group; ^#^*p* < 0.05, ^##^*p* < 0.01 compared with the hTi Group; ^†^*p* < 0.05, with the EPC/hTi Group; ^‡^*p* < 0.05, ^‡‡^*p* < 0.05 compared with the BMSC/hTi Group)

In this study, neovascularization was observed after implanting vasculogenic cells, EPC, loaded composite scaffolds into bone defects. After microangiography, blood vessels around the scaffolds were detected by Micro-CT. As indicated in Fig. 4 A, the blood vessels formation in the EPC/hTi Group and Dual/hTi Group was increased visibly. And then, quantitative analysis of BVV/TV was carried out according to the collected images. The BVV/TV value in the EPC/hTi Group (17.24 ± 2.17 %) was A significantly higher than that in the eTi Group (12.55 ± 1.60 %, *p* < 0.05) and hTi Group (12.08 ± 2.08 %, *p* < 0.05), respectively. In addition, the BVV/TV value was 22.65 ± 1.92 % in the Dual/hTi Group, which was higher when compared with that in the eTi Group (*p* < 0.01) and hTi Group (*p* < 0.01), EPC/hTi Group (*p* < 0.05) and BMSC/hTi Group (15.52 ± 1.61 %, *p* < 0.01), respectively (Fig. 4B). Generally, the microangiography detection revealed that the EPC-loaded porous scaffolds could promote neovascularization surrounding the scaffolds, which may achieve more angiogenesis inside the scaffolds. When BMSC and EPC were transplanted simultaneously, the efficiency of inducing angiogenesis was more obvious. This may be due to the synergistic effect of BMSC. BMSC have autocrine and paracrine functions, and can secrete many soluble growth factors and cytokines, as well as exosomes, which can benefit to EPC proliferation, migration and formation of blood vessels [49, 50].

Despite numerous types of 3D printed scaffolds for bone repair have been prepared and researched *in vitro* and *in vivo*, it is still challenging to design the bone tissue engineering scaffolds to induce vascularization during new bone regeneration [51]. In this study, we encapsulated BMSC and EPC in hydrogels, and then implanted them into bone defects with 3D printed porous scaffolds. Hydrogel can effectively prevent the loaded-cells from being cleaned up at the target tissue, thus ensuring the concentrated and effective distribution of the loaded-cells at the defects, and giving full play to the dual functions of osteogenesis and angiogenesis. These characteristics are of great significance for cell therapy and tissue regeneration.

### Angiogenesis- and osteogenesis-related gene expression in Bone

**Fig. 5:**
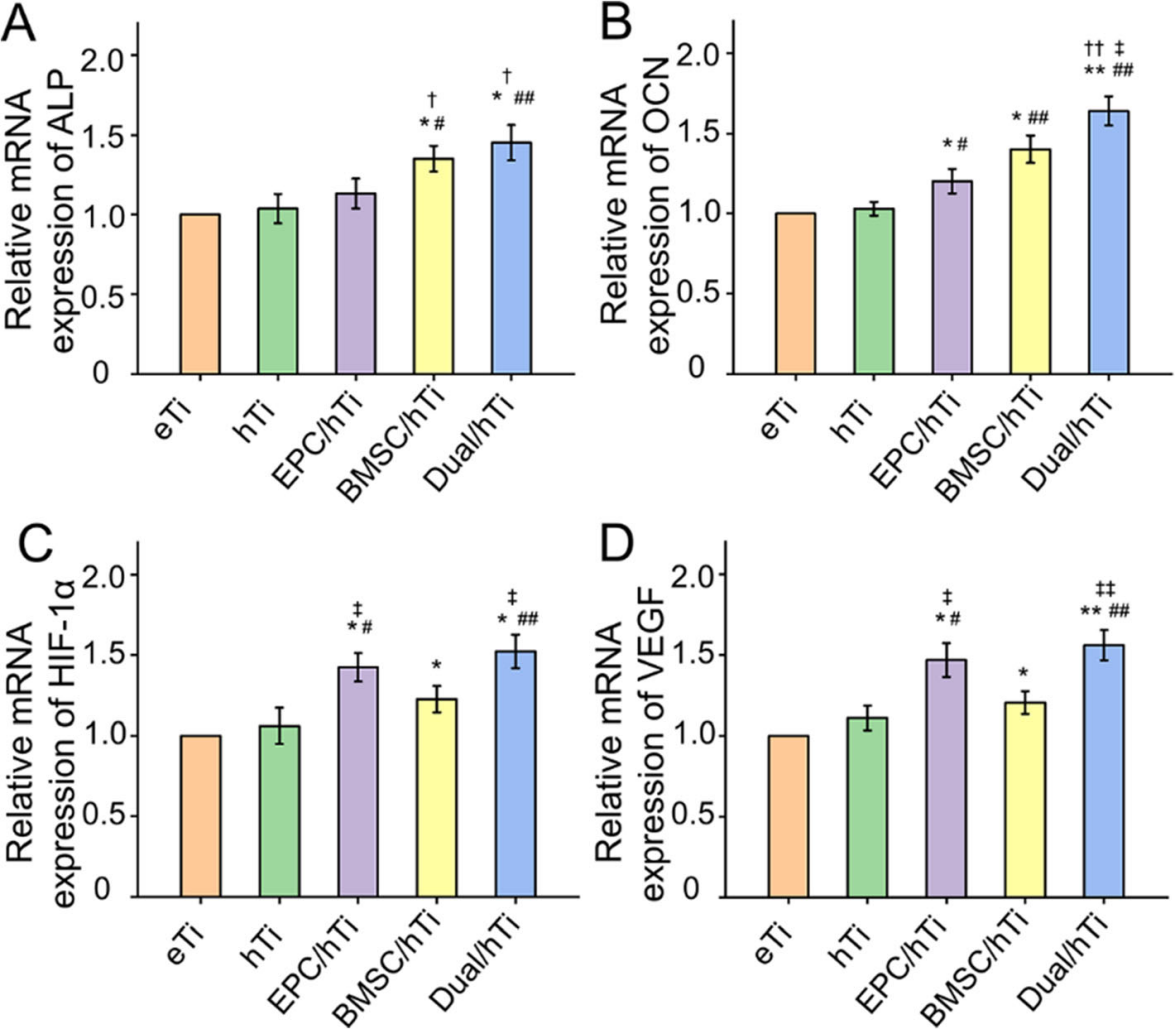
Relative mRNA expression of osteogenesis-related genes (**A**) *ALP* and (**B**) *OCN*. Relative mRNA expression of angiogenesis-related genes (**C**) *HIF-1α*, and (**D**) *VEGF in vivo* at 12 weeks after implantation (*p < 0.05, **p < 0.01 compared with the eTi Group; ^#^*p* < 0.05, ^##^*p* < 0.01 compared with the hTi Group; ^†^*p* < 0.05, ^†^*p* < 0.01 with the EPC/hTi Group; ^‡^*p* < 0.05, ^‡‡^*p* < 0.05 compared with the BMSC/hTi Group)

The expression of osteogenesis-related genes *ALP* and *OCN*, and angiogenesis-related genes *HIF-1α* and *VEGF* during the *in vivo* repair process was investigated by PCR. *ALP* is evaluated to indicate the osteogenic differentiation, and the activation of *ALP* activity and up-regulated expression of *ALP* gene is a critical event happening in early osteogenesis, which indicates the beginning of osteogenic differentiation [52]. The levels of *ALP* transcription were significantly higher in the BMSC/hTi Group and Dual/hTi Group compared with the eTi Group, hTi Group, and EPC/hTi Group (*p* < 0.05, Fig. 5A). *OCN* is always regarded as a marker of late osteogenesis to evaluate osteogenic maturation and bone formation, which is synthesized by osteoblasts [53]. The level of *OCN* expression was higher in the EPC/hTi Group, BMSC/hTi Group, and Dual/hTi Group than the eTi Group and hTi Group at 12 weeks after implantation (*p* < 0.05). In addition, the expression of *OCN* in the Dual/hTi Group was also significant up-regulated than EPC/hTi Group and BMSC/hTi Group, exhibited by Fig. 5B.

It is known that *HIF-1α* and *VEGF* is an effective factor to improve the scaffold vascularization *via* angiogenesis [15, 54]. As shown in Fig. 5C-D, the levels of angiogenesis-related gene, *HIF-1α*, was increased significantly in the EPC/hTi Group compared with eTi Group, hTi Group, and BMSC/hTi Group (*p* < 0.05). In the Dual/hTi Group, the level of *HIF-1α* was 1.52-fold, 1.43-fold, and 1.25-fold higher than the eTi Group, hTi Group, and BMSC/hTi Group (*p* < 0.05). In addition, the expression level of another angiogenesis-related gene, *VEGF*, in these groups was similar with the *HIF-1α*, namely, EPC/hTi Group and Dual/hTi Group indicated an abundant gene expression.

## Conclusions

In this study, we have constructed the 3D printed porous Ti6Al4V scaffolds filled with BMSC and EPC loaded hydrogel as a composite implant to induce angiogenesis and osteogenesis, thus promote osseointegration. Overall, the strategy of loading porous Ti6Al4V scaffolds to incorporate cells is a promising treatment for improving osseointegration.

## Data Availability

The data that support the findings of this study are available from the corresponding author (Naiqiaong Zhuo, znq0101@163.com) upon reasonable request.

## Abbreviations

3D: Three-dimensional
Ti6Al4V: Titanium alloy
BMSC: Bone marrow mesenchymal stem cells
EPC: Endothelial progenitor cells
ALP: Alkaline phosphatase
OCN: Osteocalcin
HIF-1α: Hypoxia-inducible factor 1 alpha
VEGF: Vascular endothelial growth factor
CAD: Computer aided design
Micro-CT: Microcomputed Tomography
SEM: Scanning electron microscope
CLSM: Confocal laser scanning microscope
eTi Group: Empty Ti6Al4V scaffolds
hTi Group: Composite Ti6Al4V scaffolds containing unloaded poloxamer 407 hydrogel
EPC/hTi Group: Composite Ti6Al4V scaffolds containing EPC-loaded poloxamer 407 hydrogel
BMSC/hTi Group: Composite Ti6Al4V scaffolds containing BMSC-loaded poloxamer 407 hydrogel
Dual/hTi Group: Composite Ti6Al4V scaffolds containing EPC and BMSC-loaded poloxamer 407 hydrogel
BV/TV: Bone volume/total volume
Tb.Th: Trabecular thickness
Tb.N: Trabecular number
Tb.Sp: Trabecular separation
BVV/TV: Blood vessel volume/total volume
